# Measuring behaviours for escaping from house fires: use of latent variable models to summarise multiple behaviours

**DOI:** 10.1186/s13104-015-1769-5

**Published:** 2015-12-15

**Authors:** G. B. Ploubidis, P. Edwards, D. Kendrick, on behalf of the Keeping Children Safe Study Group

**Affiliations:** London School of Hygiene and Tropical Medicine, Room 150, Keppel Street, London, WC1E 7HT UK; Division of Primary Care, School of Medicine, Tower Building, University Park, Nottingham, NG7 2RD UK

**Keywords:** Fire escape plans, Fire safety planning behaviours, Latent variable models, Methods

## Abstract

**Background:**

This paper reports the development and testing of a construct measuring parental fire safety behaviours for planning escape from a house fire.

**Methods:**

Latent variable modelling of data on parental-reported fire safety behaviours and plans for escaping from a house fire and multivariable logistic regression to quantify the association between groups defined by the latent variable modelling and parental-report of having a plan for escaping from a house fire. Data comes from 1112 participants in a cluster randomised controlled trial set in children’s centres in 4 study centres in the UK.

**Results:**

A two class model provided the best fit to the data, combining responses to five fire safety planning behaviours. The first group (‘more behaviours for escaping from a house fire’) comprised 86 % of participants who were most likely to have a torch, be aware of how their smoke alarm sounds, to have external door and window keys accessible, and exits clear. The second group (‘fewer behaviours for escaping from a house fire’) comprised 14 % of participants who were less likely to report these five behaviours. After adjusting for potential confounders, participants allocated to the ‘more behaviours for escaping from a house fire group were 2.5 times more likely to report having an escape plan (OR 2.48; 95 % CI 1.59–3.86) than those in the “fewer behaviours for escaping from a house fire” group.

**Conclusions:**

Multiple fire safety behaviour questions can be combined into a single binary summary measure of fire safety behaviours for escaping from a house fire. Our findings will be useful to future studies wishing to use a single measure of fire safety planning behaviour as measures of outcome or exposure.

Trial registration number: NCT 01452191. Date of registration 13/10/2011

## Background

Globally fire-related burns resulted in around 96,000 deaths in children and young people aged under 20 years in 2004 [1] and they are the 11th leading cause of death in children aged 1–9 years [1]. While most fire-related deaths occur in low and middle-income countries [1], they remain an important public health problem in high income countries. House fires are an important public health problem in Great Britain, with more than 43,000 occurring in 2011/12, resulting in 287 deaths and 11,300 casualties across all ages [2]. There steep social gradients in fire-related deaths in childhood; children of parents who have never worked or are long-term unemployed have fire-related death rates 38 times higher than those with parents in higher managerial or professional occupations [3]. Data from the USA demonstrates smoke alarms are associated with a two to threefold lower risk of death in house fires [4, 5] and household smoke alarm ownership has increased in Britain from 8 % in 1988 to 86 % in 2008 [2]. Given high levels of smoke alarm ownership, the emphasis of community fire safety is shifting towards the development of plans to help people escape safely from dwelling fires. In the UK, most Fire and Rescue Services offers free home fire safety checks, including help to develop a plan for escaping from a house fire [6]. More than 770,000 homes in England received a home fire safety check in 2011–12 [7].

Few published studies have evaluated the impact of interventions promoting planning for escaping from a house fire. A recent systematic review found only four studies evaluating home safety interventions that reported having or practising a plan for escaping from a house fire as an outcome measure [8–11]. Meta-analysis of these studies found home safety education was effective in increasing the proportion of families with a plan for escaping from a house fire [12], but this proportion remained relatively low post intervention, ranging across studies from 30 % [10] to 63 % [11]. Furthermore, none of the studies defined what a plan for escaping from a house fire consisted of, and all used single item questions to assess whether the family had, or had practised, a plan.

We are currently conducting a cluster randomised controlled trial evaluating a fire safety intervention delivered by children’s centres in England to families with a child aged under 3 years. The primary outcome measure for the trial is whether the family reports having a plan for escaping from a house fire. We also measure self-reported behaviours which may form elements of a plan for escaping from a house fire, such as knowing how the smoke alarm sounds, keeping a torch next to the bed, making sure exits are clear, and that the keys for the external doors and windows are readily available [13]. This paper reports the development and testing of a construct measuring parental fire safety behaviours for planning escape from a house fire.

## Methods

Home safety questionnaires often contain multiple questions about the same construct (e.g. asking families about owning a smoke alarm and about owning a fire extinguisher, two elements of the construct of fire safety). In situations such as this, statistical data reduction methods which allow multi-dimensional data sets to be made smaller, without any loss of information, may be useful. We used latent variable models as a data reduction technique to derive a single summary measure of behaviours for planning escape from a house fire.

### Sample and measures

The study sample included 1112 families from children’s centres in four sites (Nottingham, Bristol, Norwich and Newcastle, UK), who were participating in a cluster randomised controlled trial evaluating a fire safety intervention. Children’s centres were invited to participate if their catchment area had more than 50 % of under-5 year-olds living in one of the 30 % most disadvantaged super output areas in England. Families living in the catchment area were eligible if they had attended the centre in the previous 3 months; if the parents were aged 16 years or older and if they had a child aged 0–2 years. Children’s centres were stratified by trial site (4 strata) and randomly allocated within strata to treatment arms [(a) usual care, (b) fire safety injury prevention briefing and (c) fire safety injury prevention briefing plus facilitation to implement the briefing] using permuted block randomisation, with a block size of 3. The allocation schedule was produced by an independent statistician, using the Stata randomisation algorithm. Allocations were placed in sequentially numbered opaque envelopes (one set for each trial site). Information was collected on socio-demographic characteristics and fire safety behaviours of participants using a self-completed questionnaire at recruitment to the trial. Questionnaires were administered by post, telephone and face–face, depending on the method preferred by the children’s centre and the response rate. Families that completed a questionnaire were provided with a £5 gift voucher. Characteristics of participants are presented in Table 1 and fire safety behaviours in Table 2.

**Table 1:** Characteristics of participants

	n	%
Number of smokers in household		
0	759	70.1
1	226	20.9
2	97	8.0
Number of adults in household		
1	192	18.0
2	810	75.8
3 or more	67	6.2
Number of children in household		
1	542	50.6
2	351	32.8
3	121	11.3
4 or more	57	5.3
Age of youngest child		
Under 1 year	484	44.5
1–2 years	603	55.5
Mother aged 16–20		
Yes	54	5.1
No	1006	94.9
Heavy drinker in household^a^		
Yes	592	58.5
No	420	41.5
English as 1st language		
Yes	1004	91.3
No	96	8.7
Ethnic group		
White British	952	89.6
Other	111	11.4
IMD score^b^		
Mean	31.73	
Std deviation	16.64	
Minimum	2.36	
Maximum	74.8	

Not applicable and missing responses are excluded for all variables

^a^Heavy drinker defined as drinks ≥6 drinks on a typical day when they have an alcoholic drink

^b^IMD—Index of Multiple Deprivation 2010 version [27]

**Table 2:** Frequency of fire safety behaviours

	*n*	%
Has torch		
Yes	347	31.9
No	741	68.1
Knows sound of alarm		
Yes	1006	97.7
No	24	2.3
External door keys accessible		
Never	67	6.2
≤1 day/week	47	4.3
2–3 days/week	25	2.3
4–5 days/week	16	1.5
6-7 days/week	926	85.7
Window keys accessible		
Never	145	13.7
≤1 day/week	75	7.1
2–3 days/week	26	2.5
4–5 days/week	14	1.3
6–7 days/week	796	75.4
Exits clear		
Never	86	8.1
≤1 day/week	51	4.8
2–3 days/week	44	4.2
4–5 days/week	40	3.8
6–7 days/week	838	79.1
Child might hide under bed		
Strongly agree	281	32.8
Agree	27	3.2
Neither	71	8.3
Disagree	218	25.5
Strongly disagree	259	30.3
Child might hide in cupboard/wardrobe		
Strongly agree	278	32.4
Agree	23	2.7
Neither	51	5.9
Disagree	233	27.1
Strongly disagree	274	31.9

Not applicable and missing responses are excluded for all variables

### Statistical methods

A latent variable model is any model that includes unobserved random variables. The latent variable is not part of the dataset, in that it is not observed, but it is specified in order to account for any associations between the observed variables. Latent variable models combine information from different observed variables without making assumptions about units of measurement, and they also allow assessment of reliability and validity of these variables [14, 15]. To an increasing extent, latent variable modelling has been recognized as a valuable tool in epidemiological research [15] and numerous studies have employed latent variables to reduce a large number of observations and derive meaningful summaries. Other areas of application include survival analysis, meta-analysis, disease mapping, biometrical genetics, covariate measurement error models and joint models for longitudinal change and dropout.

There are two broad categories of latent variable models: *dimensional models*, where the latent variable is continuous and individuals are ranked (also called *factor analysis or item response models)*; and *discrete models*, where the latent variable is categorical and individuals are grouped (also called *latent class models*). Hybrid models where both continuous and discrete latent variables are used are also available but their use is beyond the scope of this paper.

We first used a dimensional model to create a continuous latent plan for escaping from a house fire summary measure based on a combination of binary and ordinal fire safety variables. These comprised responses from seven questions asking about fire safety relevant to planning escape from a house fire (Table 2). To find the fire safety variables most compatible to the data latent structure, we used a discrete latent variable model to derive a categorical summary measure of behaviour for planning escape from a house fire, where individuals are grouped. Due to the binary and ordinal nature of the fire safety behaviour variables, in both approaches two parameter binary and ordinal logistic link functions were used.

In the dimensional models, ‘factor loadings’ represent the strength of the association between the fire safety variable and the latent plan for escaping from a house fire summary measure (and may be interpreted as correlations). ‘Thresholds’ represent the level of the latent plan for escaping from a house fire summary measure that must be reached for a specific response (“three days a week” for example) in a categorical or ordinal fire safety variable to be given. Each individual is assigned a score on each dimension, these scores in theory range from −∞ to +∞, but in most applications the range is from −3 to 3.

In discrete models, “posterior or conditional item probabilities” represent the probability, conditional on group membership, of a category of an observed variable (having a torch for example) being given. Class probabilities specify the relative size of each class, or population prevalence of each group. Group membership is based on the observed response pattern of items under an important assumption, called the conditional or local independence assumption, which implies that the correlation among the observed variables is explained by the latent categorical variable [16].

There are a range of tests that can be used to assess how well the model fits the data with different tests for dimensional and latent class models. Statistics for these measures are given in Tables 5 and 6, along with the values which suggest a good fit of the models to the data for each approach. All models were estimated with the “complex survey design” option in the Mplus 7.0 software [17] which accommodates complex sampling designs and returns robust parameter estimates taking into account dependence due to clustering.

As an external validation criterion for our binary summary measure, we used a question from the same questionnaire in which participants were asked whether they had a plan for escaping from a house fire. Using a multivariable logistic regression model we estimated the association between parents reporting that they had a plan for escaping from a house fire and the binary summary measure, adjusting for a range of confounding variables (as listed in Table 4).

### Ethical approval

Ethical approval was provided by the NRES East Midlands Committee (Derby), reference number 11/EM/0011.

Trial registration number: NCT 01452191. Date of registration 13/10/2011

## Results

A total of 347 (32 %) of the participants reported that they had a torch beside the bed and 1006 (98 %) reported that they were familiar with the sound of their alarm. A total of 838 (79 %) participants reported that they kept their exit routes clear, 926 (86 %) that they had the external door keys and 796 (75 %) that they had window keys in accessible places on at least 6 days per week, 308 (36 %) strongly agreed or agreed that, in the event of a fire, their child might hide under the bed and 301 (35 %) that their child might hide in a cupboard or wardrobe (Table 2).

**Fig. 1:**
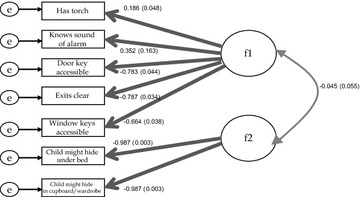
Standardised factor loadings and standard errors of confirmatory factor analysis with two dimensions *f1* and *f2*. *f1 and *f2* *circles* are latent continuous dimensions. **“*e*” *circles* represent random error in each observed indicator, or unique variance not shared with other indicators of fire safety related behaviour

First we used dimensional models to derive a continuous summary measure of behaviour for planning escape from a house fire behaviour from the seven fire safety variables. Since we had no a priori hypothesised structure for the data we estimated a sequence of exploratory models (using exploratory factor analysis) [18], with each model containing a different number of dimensions. A two-factor model (Fig. 1) was the best fitting dimensional model (see Table 5 in Appendix) which was confirmed by a confirmatory factor analysis. In the diagram f1 and f2 represent the continuous latent factors (dimensions) and the rectangles represent the observed fire safety variables.

Having external door and window keys accessible and keeping exits clear were the strongest indicators of the first plan for escaping from a house fire dimension The second plan for escaping from a house fire dimension comprised only two (highly loading) variables related to the likelihood that children may hide under a bed, or in a cupboard, in the event of a fire. The first and second plan for escaping from a house fire dimensions were not correlated with each other, suggesting their component questions were measuring different constructs. As the second dimension included only two variables unrelated to the first dimension (which included variables most relevant to planning for escape from a house fire), we excluded the variables indicating whether a child might hide from further analysis.

Secondly we used discrete models to derive a categorical summary measure of plan for escaping from a house fire behaviour. A two class model provided the best fit to the data (see Table 6 in Appendix). Table 3 presents the posterior probabilities for each fire safety variable for the two groups. The first group (‘more behaviours for escaping from a house fire) comprised 87 % of participants who were most likely to have a torch, and to report that they were aware of how their smoke alarm sounds. They were also more likely to have door and window keys accessible, and to have exits clear. For example, a typical member of this group has responded that they have a torch and are aware how their smoke alarm sounds, they have accessible door and window keys more than 2–3 times per week and have exits clear more than 4–5 times a week.

**Table 3:** Posterior probabilities derived from categorical latent variable model

	Class 1—more behaviours for escaping from a house fire	Class 2—fewer behaviours for escaping from a house fire
Has torch		
Yes	0.334	0.223
No	0.666	0.777
Knows sound of alarm		
Yes	0.982	0.944
No	0.018	0.056
External door keys accessible		
Never	0.037	0.225
≤1 day/week	0.009	0.265
2–3 days/week	0.005	0.143
4–5 days/week	0.008	0.059
6–7 days/week	0.942	0.308
Exits clear		
Never	0.051	0.276
≤1 day/week	0.005	0.328
2–3 days/week	0.021	0.177
4–5 days/week	0.034	0.061
6–7 days/week	0.889	0.158
Window keys accessible		
Never	0.095	0.403
≤1 day/week	0.033	0.310
2–3 days/week	0.010	0.114
4–5 days/week	0.011	0.028
6–7 days/week	0.850	0.144

By contrast, the second group (‘fewer behaviours for escaping from a house fire) comprised 13 % of participants who were less likely to have a torch, and less likely to be aware of how their smoke alarm sounds. They were also less likely to have door and window keys accessible, and less likely to have exits clear. A typical member of this group does not have a torch, is not aware of how their smoke alarm sounds, has accessible door and window keys less than 1 day a week, and has exits clear less than 1 day a week.

A binary summary plan for escaping from a house fire measure (‘more behaviours for escaping from a house fire’ vs. ‘fewer behaviours for escaping from a house fire’) based on the two group *latent class model* results was derived. Using a multivariable logistic regression model we estimated the association between parents reporting that they had a plan for escaping from a house fire and the binary summary measure (Table 4). After adjusting for potential confounders, participants allocated to the ‘more behaviours for escaping from a house fire group were 2.5 times more likely to report having a plan for escaping from a house fire than those in the ‘fewer behaviours for escaping from a house fire’ group (OR 2.48; 95 % CI 1.59–3.86).

**Table 4:** Odds ratios (with 95 % CIs) for reporting having a plan for escaping from a house fire according to whether allocated to the ‘more behaviours for escaping from a house fire’ group (adjusted for all confounders presented in the table)

	Odds ratio (95 % CI)
More behaviours for escaping from a house fire	2.48 (1.59–3.86)
>2 smokers in household	0.67 (0.43–1.07)
>2 adults in household	1.34 (0.52–3.44)
>2 children in household	0.35 (0.21–0.60)
Youngest child 1–2 years old	0.97 (0.77–1.22)
Mother >20 years old	0.99 (0.62–1.60)
Heavy drinker in household	0.87 (0.69–1.10)
English not first language	1.08 (0.65–1.78)
Non-white ethnic group	1.05 (0.62–1.80)
Index of multiple deprivation score	0.99 (0.98–1.01)

## Discussion

### Main findings

We have shown that multiple questions asking about behaviours for escaping from a house fire can be combined into a single binary summary measure, which provides a good fit to the data from the individual questions and it has been externally validated by comparing with a self-reported measure of having a plan for escaping from a house fire. Our findings suggest questions on having a torch beside the bed, knowing the sound of the smoke alarm, keeping exits clear and keeping door and window keys accessible appear to assess component elements of a plan for escaping from a house fire. Our findings will be useful to future studies wishing to use a single measure of planning for escape from a house fire as measures of outcome or exposure.

### Strengths and limitations

To our knowledge this is the first published paper describing the development of a measure of planning for escape from a house fire. Previous studies [8–11] have reported only single-item measures asking whether families had, or had practised a plan for escaping from a house fire. Our measure is more comprehensive than these as it uses information on range of behaviours relevant to escaping from a house fire. As we also asked families whether they had a plan for escaping from a house fire we were able to validate our newly developed measure against the reports of having a plan for escaping from a house fire, including adjusting for possible confounders. However, the reports of having an escape plan were collected on the same questionnaire as the individual items and are not therefore independent. To provide a better external validation, we would have collected reports of escape plans on a different occasion to when the questionnaire was administered.

Our multivariable model found that families with more than 2 children were less likely to report having a plan for escaping from a house fire. It is plausible that making a plan to escape from a house fire is more difficult in larger families, as there are more children (likely to be at different developmental stages) to plan for and to explain and practise the plan with. The purpose of this analysis was to provide some validation of our summary measure, not to explore factors associated with having a plan to escape a house fire. This finding should therefore be interpreted with caution and requires replication in other studies.

Our behaviours for planning escape from a house fire were self-reported, with most questions asking about the frequency of behaviours and one question asking about knowledge of how smoke alarms sound. Validating these, for example, by home observations, is not possible, and to our knowledge there are no published studies reporting reliability or validity of these measures [19–26]. Two studies have reported some measures of reliability and validity of self-reported plans for escaping from a house fires. The first found a high level of agreement (91 %; no kappa coefficients reported) to repeat administration (over 14–24 days) of a single item question (Do you have a plan for escape from the home in the event of a fire?) as part of a general home safety survey amongst 35 parents of children aged 1–4 years [21]. This question is very similar to that asked in our trial, suggesting the test–retest reliability of our question about having a plan for escaping from a house fire may be reasonable. An Australian study reported no significant difference in reported prevalence of having a fire evacuation plan on a telephone survey and home observation, although they did not report the percentage agreement, kappa coefficients, sensitivity, specificity or predictive values [20]. It is possible that parents in our study over-reported behaviours they perceived to be socially acceptable. This could affect our findings if over-reporting varied substantially between the safety behaviours comprising our summary binary measure, although there is no reason to believe some of these safety behaviours are much more socially acceptable than others.

Our study was restricted to families with young children attending children’s centres in England. Children’s centres provide integrated early years health, social care and education to families living in disadvantaged areas. So, whilst our findings are applicable to families whose children are at greater risk of fire-related injury, they may not be generalisable to families with older children, more advantaged populations, to those from low and middle-income countries, or to planning for escaping from a house fires amongst other vulnerable populations such as the elderly or disabled.

The distribution of the first plan for escaping from a house fire dimension was highly skewed and resembled a mixture of distributions rather than a continuum. Using this distribution even with appropriate link functions as an outcome in predictive models would have been potentially biased since the two suspected mixture components may be associated with different sets of predictors. We therefore decided to use a categorical latent variable model to create our single summary measure.

### Implications for research and practice

Our findings will be useful for future studies in high income countries where planning for escape from a house fire will be similar to that in the UK and for populations comprising families with small children. Such studies can use our questions to develop a single summary measure of fire escape planning behaviour. Many injury prevention interventions are evaluated using tools which measure multiple safety behaviours. The methods we describe could be used to develop single outcome measures for evaluation studies, or single measures of exposures for observational studies. This may be helpful in minimising the risk of “spuriously” significant findings resulting from multiplicity (statistical significance testing of multiple outcomes or exposures). Our findings will be of use to injury prevention practitioners who help families to develop plans for escaping from a house fire, as our questions could be used in home fire safety risk assessments or home safety check lists, and could be used by practitioners to assess the impact of their work on planning escape from house fires.

## Conclusion

Multiple fire safety behaviour questions can be combined into a single binary summary measure of planning for escape from a house fire. Our findings will be useful to future studies wishing to use a single measure of behaviours for planning for escape from a house fire as measures of outcome or exposure.

## Figures and Tables

**Fig. 2:**
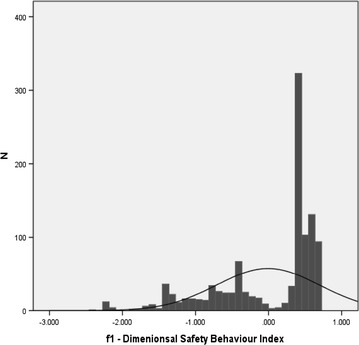
Histogram of dimensional safety behaviour summary index (*f1*)

## Appendix

See Tables 5 and 6 and Fig. 2.

**Table 5:** Assessment of fit of dimensional latent variable models

	*X* ^2^	*p*	CFI	TLI	RMSEA
Exploratory factor analysis—1 factor	708.249	0.001	0.986	0.98	0.176
Exploratory factor analysis—2 factors	15.236	0.293	1	1	0.012
Confirmatory factor analysis—2 factors	9.998	0.762	1	1	0.001

*CFI* comparative Fit Index, values >0.95 indicate food fit

*TLI* Tucker Lewis Index, values >0.95 indicate good fit

*RMSEA* root mean square error of approximation, values <0.06 indicate good fit

**Table 6:** Assessment of fit of categorical latent variable models—latent class analysis

	−2LL	AIC	SSA-BIC	Entropy
1 Class	−3139.53	6307.05	6332.76	1
2 Classes	−2950.85	5959.70	6012.96	0.84
3 Classes	−2875.93	5839.86	5920.68	0.77
4 Classes	−2820.53	5759.07	5867.43	0.84

*−2LL* minus twice the loglikelihood, smaller values indicate better fit

*AIC* akaike information criterion, smaller values indicate better fit

*SSA BIC* sample size adjusted bayesian information criterion, smaller values indicate better fit

*Entropy* values close to 1 indicate more reliable classification (lower classification error)
